# Supplementary material for: Association between oral microbiome and seven types of cancers in East Asian population: a two-sample Mendelian randomization analysis
Source: Front Mol Biosci. 2023 Nov 21;10:1327893. doi: 10.3389/fmolb.2023.1327893 (PMC10702768; doi:10.3389/fmolb.2023.1327893)
Supplement: Supplementary file 1 [file DataSheet1.ZIP › Supplementary Materials/MR plots for tongue/tongue═╝/Lung cancer/pheno.3114_to_lung cancer_forest.pdf]

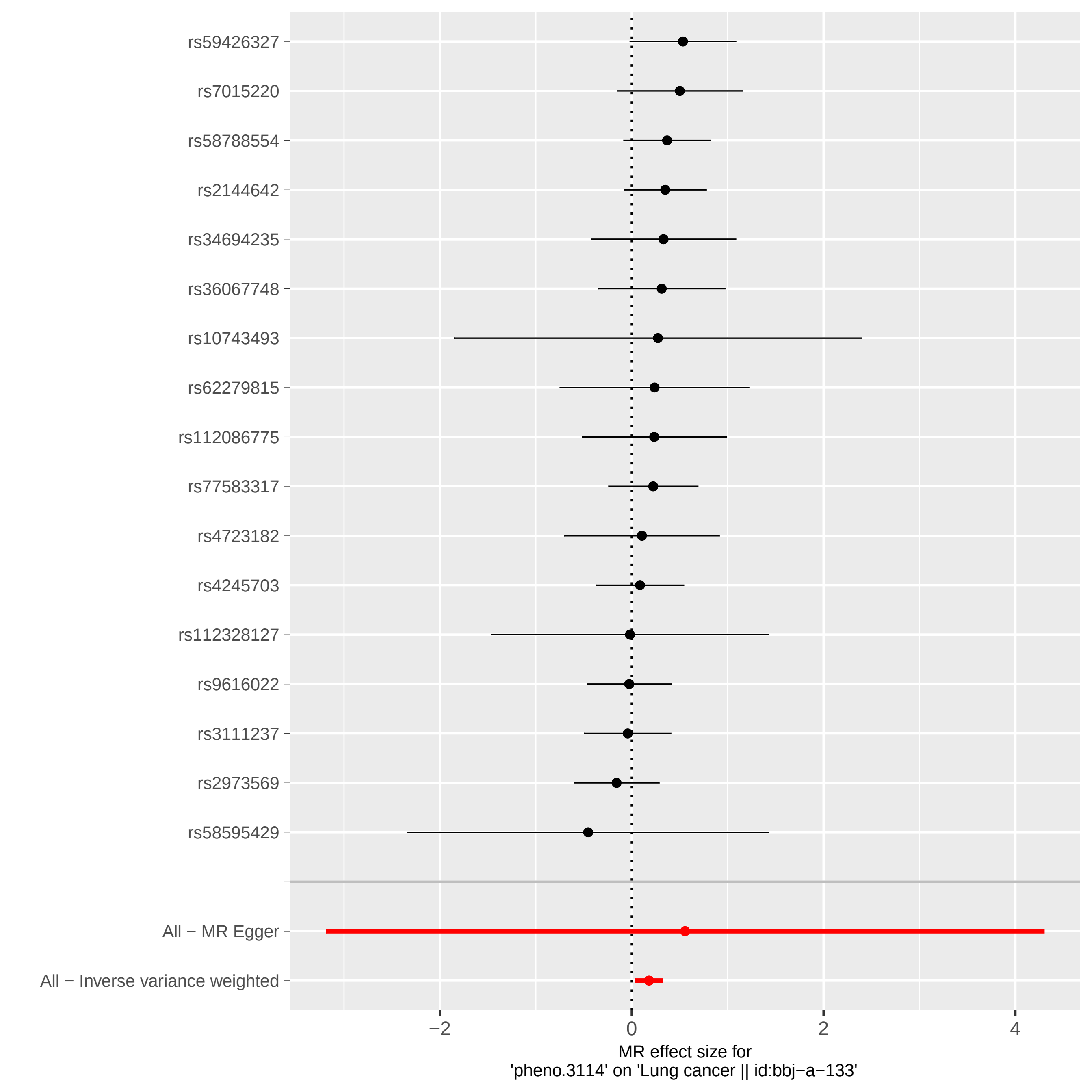
rs59426327
rs7015220
rs58788554
rs2144642
rs34694235
rs36067748
rs10743493
rs62279815
rs112086775
rs77583317
rs4723182
rs4245703
rs112328127
rs9616022
rs3111237
rs2973569
rs58595429
All − MR Egger
All − Inverse variance weighted
−2
0
2
4
MR effect size for
'pheno.3114' on 'Lung cancer || id:bbj−a−133'
